# Oxidative Stress Induced by Polymyxin E Is Involved in Rapid Killing of* Paenibacillus polymyxa*

**DOI:** 10.1155/2017/5437139

**Published:** 2017-02-22

**Authors:** Zhiliang Yu, Yuyi Zhu, Wangrong Qin, Jianhua Yin, Juanping Qiu

**Affiliations:** College of Biotechnology and Bioengineering, Zhejiang University of Technology, Hangzhou, China

## Abstract

Historically, the colistin has been thought to kill bacteria through membrane lysis. Here, we present an alternative mechanism that colistin induces rapid* Paenibacillus polymyxa *death through reactive oxygen species production. This significantly augments our understanding of the mechanism of colistin action, which is critical knowledge toward the yield development of colistin in the future.

## 1. Introduction

Polymyxin E, also called colistin, is biosynthesized by* Paenibacillus polymyxa* [1]. As a nonribosomal cyclic lipopeptide antibiotic, it is recognized as one of the final options of antibiotic therapy for multidrug-resistance (MDR) bacteria resistant to almost all other currently used antibiotics [2]. Development of MDR pathogens against common antibiotics is increasing worldwide at an alarming rate. Although the concerns on colistin nephrotoxicity and neurotoxicity still remain, the use of colistin in many health care centers has been reevaluated by medical community [3]. Colistin bears several positively charged 2,4-diaminobutyric acids (Dab) which readily bind to the negatively charged lipid A on the cell membrane via electrostatic attraction. Upon initial binding, colistin displaces the divalent cations (Ca^2+^ and Mg^2+^) on the membrane and inserts its hydrophobic segments into membrane, thus weakening the packing of adjacent lipid A and causing membrane expansion [4, 5]. As a result of destabilized area formation [6, 7], colistin destroys the physical integrity of phospholipid bilayer on the membrane and causes membrane lysis [8]. It is generally believed that colistin only kills the Gram-negative bacteria by inducing membrane lysis [9, 10].

Recent report showed an alternative mechanism of colistin against Gram-negative bacteria [11]. Colistin stimulates the production of highly deleterious hydroxyl radicals (^•^OH), leading to Gram-negative bacteria cell death. However, the mechanism underlying ^•^OH formation is not very clear yet. One explanation is that the mechanism of ^•^OH formation induced by bactericidal antibiotics is probably the end product of oxidative damage to cell involving generation of superoxide (O_2_^−^), destabilization of Fe-sulfur (Fe-S) clusters, and stimulation of Fenton reaction [12]. Upon exposure to antibiotics, a modest surge in consumption of NADH generated from NAD^+^ via tricarboxylic acid (TCA) cycle likely induces a burst in O_2_^−^ generation via accelerated respiratory chain [12, 13]. Furthermore, antibiotics induce cellular redox state alterations, and these toxic perturbations may contribute to the lethality of antibiotics [14, 15]. Fe-S clusters are highly susceptible to oxidative attack from O_2_^−^, leading to the release of ferrous irons (Fe^2+^). On the other side, O_2_^−^ will be converted to H_2_O_2_ by superoxide dismutases (SOD) present in the cell. Subsequently, H_2_O_2_ will oxidize Fe^2+^ to ferric iron (Fe^3+^), along with ^•^OH formation, which is called Fenton reaction [16]. All these biologically oxidative species including O_2_^−^, H_2_O_2_, and ^•^OH are called reactive oxygen species (ROS). When the concentration of ROS reaches uncontrollable level, it will result in oxidative damage to DNA, lipids and proteins, and eventually cause cell death [17, 18]. In this process, oxidative damage of Fe-S clusters is a key source of Fe^2+^ driving ^•^OH formation and the elevated intracellular Fe^2+^ has been shown to mediate cellular damage not only directly but also via additional ^•^OH formation [19]. The inactivation of intracellular Fe-S cluster will damage the Fe-S dependent proteins, especially Fe-S dependent dehydratases such as dihydroxy-acid dehydratase (DHAD). The damaged DHAD can be repaired by protein YggX (a member of SoxRS regulon) and di-iron protein YtfE in the presence of Fe^3+^ whose introduction is strongly triggered by ferric uptake regulator (Fur) [20].

Current understanding of the antibacterial mechanisms of colistin mainly focuses on Gram-negative bacteria. The knowledge on its bactericidal activity against the Gram-positive bacteria is extremely little. Yet, few studies showed that colistin can also kill the Gram-positive bacteria [10], but the detailed mechanism is not clear [21, 22]. In this study, we demonstrated that colistin can induce the ROS accumulation in its producer* P. polymyxa*, a Gram-positive bacterium, probably playing a key role in rapid killing of Gram-positive bacterial cells. Since colistin is produced by* P. polymyxa*, its bactericidal activity against its producer would potentially repress its accumulation during fermentation. Therefore, our findings not only enrich our understanding of the bactericidal activity of colistin against Gram-positive bacteria but also help to improve its fermentation output in the future.

## 2. Materials and Methods

### 2.1. Strain and Culture Condition [10]

*P. polymyxa *used in this work was supplied by Zhejiang Qianjiang Biochemical Co., Ltd., China, and frozen at −80°C in our lab. Unless otherwise stated,* P. polymyxa* was grown on solid medium including 10 g/L of beef extract, 15 g/L of peptone, 10 g/L of glucose, 2 g/L of yeast extract, 3 g/L of NaCl, and 18 g/L of agar at 30°C for 2 d. Then, a ring of* P. polymyxa* was inoculated to 50 mL of broth medium (solid medium without agar) in 250 mL flask for incubation at 30°C for 18 h with a shaking at 200 rpm.

### 2.2. Treatment of* P. polymyxa* by Colistin [10]

Upon broth incubation, the cells were collected by centrifugation at 5,000*g* for 10 min. After washing once with fresh broth medium, the cells were resuspended in fresh broth medium with appropriate volume to make a final cell concentration of about 10^7^ colony-forming units per milliliter (CFU/mL). Then, unless otherwise specified, colistin with a final concentration of 1.6 × 10^5^ U/mL (supplied by Zhejiang Qianjiang Biochemical Co., Ltd., China) together with or without radical scavenging compound was added to cell solution and the mixture was incubated for various times at 30°C with a shaking at 200 rpm. One unit is equal to 0.0418 *μ*g of colistin.

### 2.3. Detection of Total Plate Count

After colistin treatment with or without radical scavenging compound, the mixture was centrifuged at 4,000*g* for 5 min. After washing twice with fresh broth medium, the cells were resuspended and appropriately diluted in fresh broth medium. Then, appropriate volume of cells was spread to solid medium for growth. After cultivation at 30°C for 2 d, CFU was counted.

### 2.4. Measurement of Intracellular Levels of ROS

The intracellular levels of ROS were measured using a reactive oxygen species assay kit (Beyotime, Jiangsu, China) according to manufacturer's protocol. In general, 1 mL of bacterial cells (10^7^ CFU/mL) with or without treatment of colistin was subjected to 30 *μ*L of 10 *μ*M 2,7-dichlorofluorescein diacetate (DCFH-DA). After treatment at 37°C for 20 min, the cells were collected with centrifugation at 4,000*g* for 5 min and washed twice with fresh broth medium. After resuspension in 1 mL of fresh broth medium, 200 *μ*L of the cells were analyzed by a multimode reader (SpectraMax M2, USA) with excitation and emission of 488 and 525 nm, respectively.

### 2.5. Determination of Lipid Peroxidation

Lipid peroxidation was evaluated based on the production of malondialdehyde (MDA) which was quantified using thiobarbituric acid assay [24]. In brief, 1 mL of bacterial cells (10^7^ CFU/mL) with or without treatment of colistin was collected with centrifugation at 4,000*g* for 5 min and washed twice with fresh broth medium. Then, the cells were resuspended in 100 *μ*L of breaking solution containing 5% SDS and 250 mM EDTA for incubation at 37°C for 30 min. Next, according to manufacturer's protocol, the MDA in mixture was detected using an MDA assay kit (Beyotime, Jiangsu, China) and analyzed by a multimode reader (SpectraMax M2, USA) at a wavelength of 532 nm.

### 2.6. Genomic DNA Extraction and Analysis

One milliliter of bacterial cells (10^7^ CFU/mL) was treated with or without colistin for various times as needed. Then, the cells were collected with centrifugation at 4,000*g* for 5 min and washed twice with fresh broth medium. Next, the cells were subjected to lysis buffer containing 125 *μ*L of 500 mM EDTA with pH 8.0, 20 *μ*L of 10 mg/mL lysozyme and 1 *μ*L of 10 mg/mL RNA enzyme for incubation at 37°C for 30 min. Subsequently, 70 *μ*L of 10% SDS together with 5 *μ*L of 10 mg/mL protease K were added to lysis solution for further incubation at 55°C for 30 min. Then, the sample was completely mixed with 70 *μ*L of NaCl. After ice-bath for 30 min, the mixture was centrifuged at 12,000*g* for 30 min at 4°C. The supernatant was mixed with an equal volume of Tris-phenol. After centrifugation at 12,000*g* for 10 min at 4°C, the supernatant was mixed with two volume of ice-cold absolute ethanol for precipitation at −20°C for 20 min. After centrifugation at 12,000*g* for 10 min at 4°C followed by washing with 500 *μ*L of 75% ice-cold ethanol, the DNA pellets were air-dried and dissolved in around 50 *μ*L of ddH_2_O. After concentration balance, the DNA samples were run on a 1% agarose gel and visualized under UV light.

### 2.7. Cloning of* P. polymyxa* Genes

The primers listed in Table 1 were designed using Primer Premier 5.0 software based on the reference gene sequences from* P. polymyxa* E681 (GenBank accession number CP000154) and* P. polymyxa* SC2 (GenBank accession number CP002213) and synthesized by Invitrogen (Carlsbad, California, USA). PCR was performed in a final volume of 50 *μ*L containing 2 ng* P. polymyxa* genomic DNA, 100 nM each of primers, 62.5 *μ*M each of dNTPs, 50 mM KCl, 10 mM Tris-HCl, 1.5 mM MgCl_2_, and 1 U* Taq* polymerase (Amersham Biosciences, Piscataway, USA). PCR amplification consisted of denaturation at 95°C for 5 min, followed by 35 cycles of 30 s at 95°C, 30 s at 55°C, 2 min at 72°C, and a final extension step at 72°C for 10 min. At the end of the reaction, the reaction mixture was cooled to 4°C to await further manipulations. The PCR products were resolved on 1.0% agarose gel for electrophoresis, and the product size was checked on the gel stained with ethidium bromide under UV. After size confirmation, the target DNA in gel was extracted using MiniBEST Agarose Gel DNA Extraction Kit (TaKaRa, Dalian, China) and cloned into pMD19-T simple vector (TaKaRa, Dalian, China). Finally, each gene sequence was determined after sequencing (Sangon, Shanghai, China).

### 2.8. Analysis of Gene Expression Using Quantitative Real-Time PCR (qRT-PCR)

To measure the gene expression, qRT-PCR was used to amplify cDNA products reversely transcribed from mRNA [25, 26]. In brief, the bacterial cell was harvested through centrifugation at 5,000*g* for 5 min and the total RNA was extracted using an RNAiso Plus kit (Sangon, Shanghai, China). RNA integrity was determined based on the OD_260 nm_/OD_280 nm_ ratio (>1.95), and 500 ng of DNA-free RNA with high-quality was reversely transcribed to cDNA in a 10 *μ*L volume using PrimeScript™ RT Master Mix (Perfect Real-Time) kit. After appropriate dilution, the cDNAs were used to amplify the target gene fragments from 100 bp to 120 bp with primer sets (Table 2) by using the SYBR green* Premix Ex Taq*™ (Tli RNaseH Plus) kit. PCR was run on CFX Connect Real-Time System (Bio-Rad, Hercules, CA) based on an amplification protocol consisting of an initial denaturation at 95°C for 10 min, followed by 40 cycles of denaturation at 95°C for 15 s and annealing/elongation at 60°C for 30 s. Immediately after the final PCR cycle, a melting-curve analysis was made to determine the reaction specificity based on the observed melting temperature from product. Unless otherwise specified, all the kits above were purchased from TaKaRa Bio. Inc. (Dalian, China).

The cycle threshold (*C*_*T*_) for each PCR was determined using STATVIEW software which automatically set the threshold signal at the log phase of amplification curve. Several dilutions of each cDNA sample were assayed for gene of interest in order to obtain a linear regression between the *C*_*T*_ values (ranging from 20 to 35 cycles) and the log of cDNA. The amplification efficiency of gene was retrieved from the slope of that linear regression according to the formula *E* = 10^(−1/slope)^. The 116 bp of housekeeping 16S rRNA gene fragment was amplified and treated as the internal control to verify that there were equal amounts of target cDNA in all samples. The relative expression of the target gene compared to that of the reference 16S rRNA gene was calculated by comparative *C*_*T*_ method [27].

### 2.9. Detection of Fe^3+^ Based on Ferric-Xylenol Orange Formation

Xylenol orange (XO) can combine with Fe^3+^ to form purplish Fe^III^XO. Unless otherwise stated, Fe^III^XO agar assay was used for detection of the concentration of Fe^3+^ [28] as follows: (1) prepare 20 mL of working solution containing 800 *μ*L of 1 M H_2_SO_4_, 2 mL of 1 M D-Sorbitol, 3 mL of 1 mM XO, and 14.2 mL of ddH_2_O; (2) make 50 mL of 2% agar solution and dissolve the mixture completely at 100°C for 5 min; (3) mix working solution with agar solution and pour into glass Petri dish to make agar plate; (4) make circular wells on agar plate with a hole puncher whose diameter is 6 mm; (5) add 50 *μ*L of detection solution to each well and wait for 60 min at room temperature for color change; (6) visualize the Fe^III^XO formation and measure the size of purplish red halo. A series of standard Fe^3+^ solutions with different concentrations were used to extract the correlation between Fe^3+^ concentration and diameter of the formed purplish red halo.

### 2.10. Data Analysis

All data were presented as mean ± standard error and tested for statistical significance based on analysis of variance (ANOVA) followed by Dunnett's post hoc test using StatView 5.0 program. When the probability (*p*) was less than 0.05, 0.01, and 0.001, the values were considered significantly (*∗*), very significantly (*∗∗*), and extremely significantly (*∗∗∗*) different, respectively.

## 3. Results

### 3.1. Rapid Killing of* P. polymyxa* Induced by Colistin

**Figure 1:**
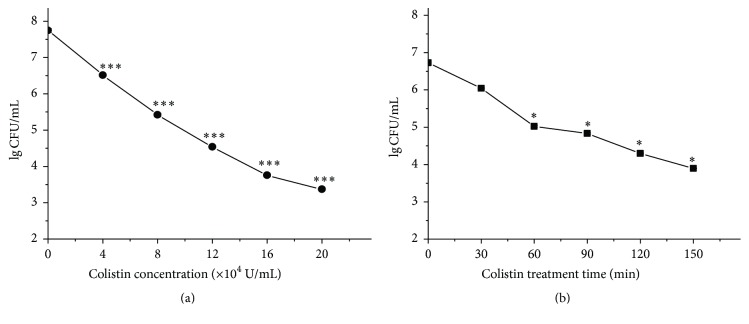
Colistin-induced rapid killing of* P. polymyxa*. (a) Colistin with various concentrations for 2 h; (b) colistin at 8 × 10^4^ U/mL for various times. After treatment, cell solution with appropriate dilution was plated for cultivation and the CFU was counted.

Bactericidal activity of colistin against* P. polymyxa* was tested based on total plate count assay. Results in Figure 1(a) show that colistin with concentration from 4 × 10^4^ U/mL to 2 × 10^5^ U/mL all causes extremely significant decrease of the cell survival. Besides, its bactericidal activity is positively correlated with its concentration. As the colistin concentration increases, the cell survival correspondingly decreases. In addition, data in Figure 1(b) indicate that the bactericidal activity of colistin against* P. polymyxa* is time-dependent. The longer the treatment, the stronger the bactericidal activity. These findings, together with others' studies [21, 22], expand the bactericidal activity of colistin to Gram-positive bacteria.

### 3.2. ROS Accumulation and Oxidative Stress-Induced Damage in Colistin-Exposed* P. polymyxa* Cells

**Figure 2:**
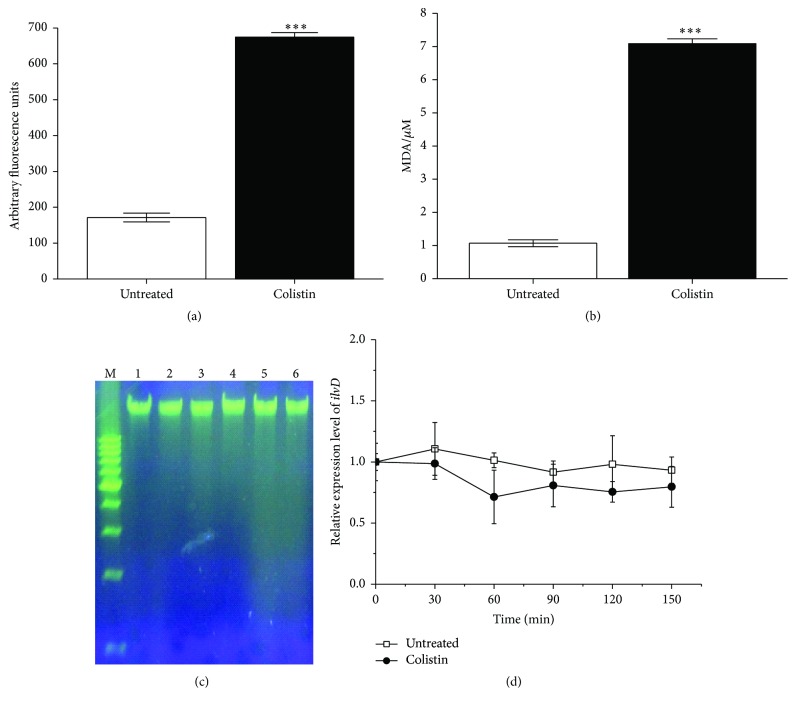
ROS accumulation in* P. polymyxa* and oxidative stress-induced damage to cell. (a) Fluorescence signal of DCF-DA converted from DCFH-DA due to oxidation, (b) lipid peroxidation production of MDA, (c) visualization of genomic DNA fragmentation, and (d) Relative expression level of* ilvD *encoding for DHAD. For panels (a), (b), and (d), cells were treated with or without 1.6 × 10^5^ U/mL colistin for 2 h. The data are representative of three independent experiments. Points represent the means and bars represent the standard deviation of triplicate samples. For panel (c), lanes 1 to 6 refer to genomic DNA from* P. polymyxa* treated with 0, 4 × 10^4^ U/mL, 8 × 10^4^ U/mL, 1.2 × 10^5^ U/mL, 1.6 × 10^5^ U/mL, and 2.0 × 10^5^ U/mLof colistin, respectively; M represents molecular mass marker.

Using the dye DCFH-DA (autofluorescence unit is 60.678) which could be oxidized to DCF-DA by ROS [29], we specifically detected the ROS accumulation in bacterial cells treated with colistin. Compared to the control, the fluorescence signal significantly increases in the presence of colistin (Figure 2(a)), suggesting that colistin could induce the ROS accumulation in* P. polymyxa*. It has been revealed that the oxidative stress can drive the oxidization of lipid in cell membrane and degradation of DNA [30]. Our results in Figure 2(b) also show that the cells subjected to 8 × 10^4^ U/mL colistin yield around 7 *μ*M of MDA, an oxidized product of polyunsaturated fatty acid, extremely and significantly higher than 1 *μ*M of MDA in the cells without colistin. Additionally, analysis of genomic DNA in agarose gel (Figure 2(c)) indicates that colistin induces the genomic DNA degradation in* P. polymyxa* accompanied by an increase in DNA smear with the increase in colistin concentration. As Fe-S cluster protein, DHAD is extremely sensitive to oxidative stress [20].  Figure 2(d) shows that the relative expression of* ilvD* (encoding DHAD) is downregulated by colistin. Taken together, our results suggest that colistin can induce ROS accumulation in* P. polymyxa*, subsequently killing Gram-positive bacteria cells by triggering diverse damage.

### 3.3. Delay of Colistin-Induced Killing of* P. polymyxa* by Scavenging ROS

**Figure 3:**
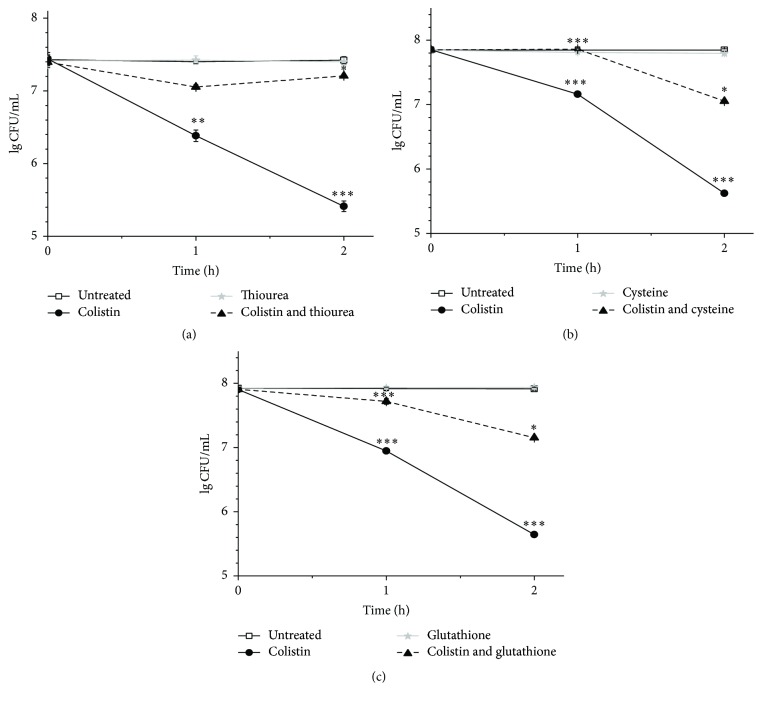
Delay of colistin-induced killing of* P. polymyxa* by scavenging ROS with 100 mM thiourea (a), 100 mM cysteine (b), or 100 mM glutathione (c).

Considering the observation that colistin can induce ROS formation for killing of* P. polymyxa*, we sought to rescue colistin-treated cells by scavenging ROS. Accordingly, thiourea was first used as a potent scavenger to particularly quench ^•^OH, a type of ROS [31].  Figure 3(a) shows that colistin alone causes a pronounced decrease of LgCFU/mL from around 7.4 to 5.4 within 2 h, while extra addition of thiourea increases the LgCFU/mL of* P. polymyxa* to 7.2 at 2 h, close to the level without colistin. As expected, the treatment with thiourea alone has no obvious effect on LgCFU/mL of* P. polymyxa*. These results indicate that thiourea is able to extremely and significantly relieve the colistin-mediated mortality to* P. polymyxa*. We further examined both glutathione (Figure 3(b)) and cysteine (Figure 3(c)), another two types of ROS sequesters, to rescue colistin-treated* P. polymyxa*. It was found that these two ROS sequesters yield overall similar results as thiourea. Notably, application of either glutathione or cysteine can increase the survival close to the level without colistin at 1 h. All these findings demonstrate that colistin stimulates the production of highly deleterious ROS in Gram-positive bacteria, which ultimately contributes to cell death. ROS sequesters can remarkably protect cells from the colistin-mediated killing.

### 3.4. Protective Response of* P. polymyxa* to Colistin-Induced Oxidative Stress

**Figure 4:**
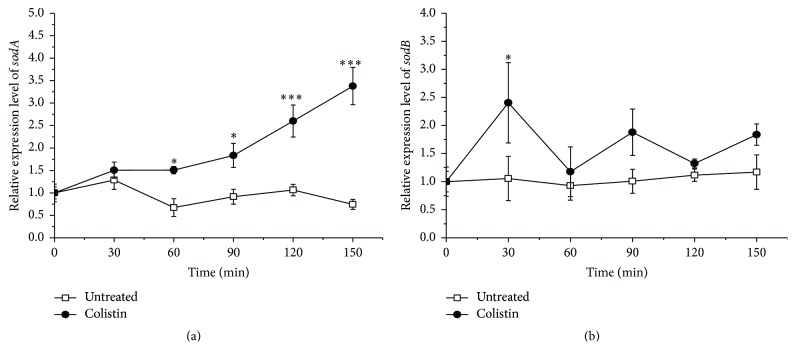
Effect of colistin on relative expression level of* sodA* (a) and* sodB* (b) encoding for Mn-SOD and Fe-SOD, respectively, in* P. polymyxa*.

Typically, SOD plays a key role for cells to survive under oxidative stress [32, 33]. Genome analysis reveals that* P. polymyxa* appears to have Mn-SOD and Fe-SOD, encoded by* sodA* and* sodB*, respectively. Mn-SOD synthesis is stimulated by O_2_^−^ [34]. Fe-SOD is important for protecting cytoplasmic enzyme such as DHAD from oxidative damage [35]. Our results in Figure 4 display the changes in the relative expression level of* sodA* and* sodB* in* P. polymyxa*. The* sodA* expression is significantly stimulated by the addition of colistin and keeps increasing with the treatment time. Besides, it gives a near 3.5-fold increase relative to the treatment without colistin at 150 min. In contrast, the* sodB* expression is also overall increased but fluctuated following addition of colistin, which is highly correlated with the change in the* ilvD* expression (Figure 2(d)). Most probably, the inactivation of DHAD in transcriptional level by colistin-induced oxidative stress needs transient activation of Fe-SOD for protection, a consistence with the report [35].

**Figure 5:**
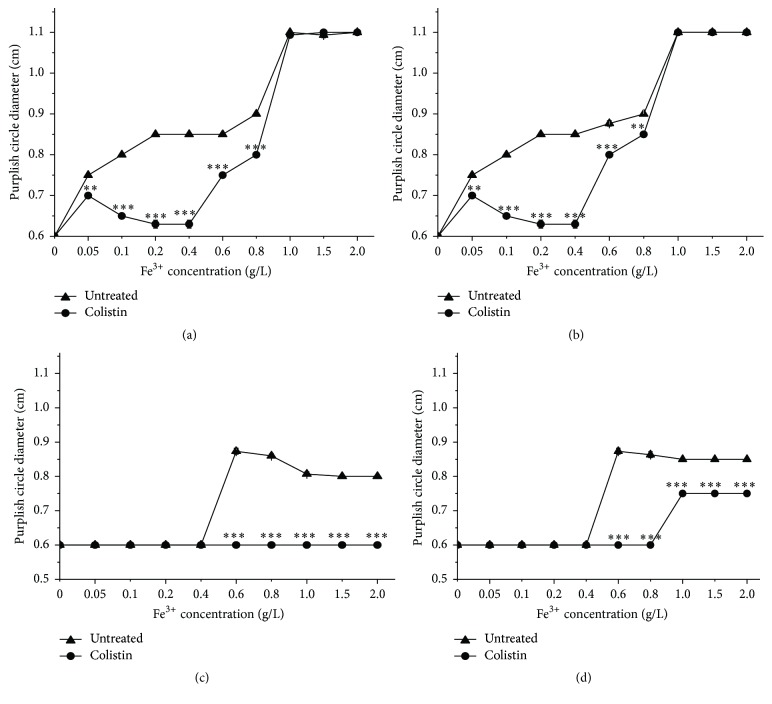
Changes in diameter of purplish red Fe^III^XO halo formed from supernatant (a and b) and cell pellet (c and d) of colistin-treated* P. polymyxa* with various FeCl_3_. Before detection of purplish red Fe^III^XO halo formation, there is H_2_O_2_ treatment for (b) and (d), but not for (a) and (c).

In addition to SOD, iron, and iron-associated proteins are also very important for cells to response with oxidative stress. Fe^2+^ is involved in extremely deleterious ^•^OH formation via the Fenton reaction. Conversely, Fe^3+^ can be utilized to repair the damaged Fe-S cluster proteins. Therefore, iron acquisition and metabolism are strictly regulated for cells to survive in oxidative stress. There is increasing evidence to reveal the coordination between regulation of iron homeostasis and defense of oxidative stress [36]. We prepared fresh broth media with different concentrations of Fe^3+^ for treatment of cells with colistin and subsequently using Fe^III^XO-formation agar assay [28] to determine the iron distribution both in supernatant solutions and inside cells.  Figure 5(a) shows that the diameters of the purplish red hole formed by the supernatant collected from the solution in the absence of colistin (untreated), which expectedly keep increasing with higher Fe^3+^ concentration from 0 to 1 g/L and saturated at Fe^3+^ > 1 g/L, indicating the diameter of purplish red hole is positively sensitive to Fe^3+^ at detectable concentration for assay. Interestingly, treatment with colistin alone causes the diameter of purplish red hole derived from Fe^3+^ ranging from 0.05 to 1 g/L significantly smaller relative to the untreated one, suggesting that colistin causes the disappear of partial Fe^3+^ from solution (Figure 5(a)). Presumably, due to the saturation caused by Fe^3+^ from 1 to 2 g/L, there is no clear discrepancy in diameter of purplish red hole between colistin absent and present. There are two possible reasons for disappear of Fe^3+^ under colistin exposure: (1) it has been turned to Fe^2+^; (2) it has been absorbed into cells. To test the first possibility, we additionally used H_2_O_2_ to oxidize the possibly formed Fe^2+^ in colistin-treated supernatant before detection and then measured Fe^3+^. The data in Figure 5(b) show that H_2_O_2_ treatment gives almost the similar results as the H_2_O_2_ untreated (Figure 5(a)) and does not obviously increase the diameter of purplish red hole formed from the colistin-subjected supernatant, thus demonstrating that the disappeared Fe^3+^ has been mostly assimilated into cells. Exceptionally, H_2_O_2_ treatment yields the marginally elevated diameter of purplish red hole relative to H_2_O_2_ absent at Fe^3+^ from 0.6 to 0.8 g/L, suggesting that very small portion of Fe^2+^ has been either formed from Fe^3+^ or released from cells when exposed to colistin. We next collected the cells for ultrasonication and then detected the released intracellular Fe^3+^ concentration in supernatant solution after centrifugation. As shown in Figure 5(c), there is no detectable and almost constant intracellular Fe^3+^ from colistin-absent cells when exposed to Fe^3+^ from 0 to 0.4 g/L and 0.4 to 2 g/L, respectively, suggesting that treatment of Fe^3+^ only with high enough concentration causes the uptake of detectable Fe^3+^ into cells. Surprisingly, there is constantly no detectable intracellular Fe^3+^ from colistin-treated cells when exposed to Fe^3+^ from 0 to 2 g/L. The missing of the assimilated Fe^3+^ in colistin-treated cells probably contributes to two reasons: (1) colistin is able to induce the conversion of intracellular Fe^3+^ to Fe^2+^; (2) colistin could result in the conjugation of intracellular Fe^3+^ with Fe^3+^-binding proteins. Next, we additionally used H_2_O_2_ to oxidize the possibly formed Fe^2+^ released from colistin-treated cells and detected the total Fe^3+^ concentration.  Figure 5(d) shows that H_2_O_2_ treatment to colistin-free sample gives almost the same diameter of purplish red hole as the H_2_O_2_ untreatment (Figure 5(c)), thus suggesting that there is no obviously detectable Fe^2+^ in colistin-free cells. In contrast, H_2_O_2_ treatment to colistin-present sample with Fe^3+^ from 1.0 to 2 g/L does additionally yield the detectable diameter of purplish red hole relative to the H_2_O_2_-untreated one, thus demonstrating colistin treatment is able to induce oxidative stress and correspondingly stimulate the conversion of absorbed Fe^3+^ to Fe^2+^ inside cells. On the other hand, additional H_2_O_2_ treatment to colistin-present sample with Fe^3+^ less than 1.0 g/L is unable to create the detectable purplish red hole. Besides, when colistin-treated cells are subjected to Fe^3+^ ranging from 1.0 to 2 g/L, the diameter of purplish red hole from Fe^3+^ oxidized from intracellular Fe^2+^ by H_2_O_2_ is smaller than the one from colistin-absent sample. All these findings support the fact that colistin can induce cells to couple iron with binding proteins for iron sequestering or Fe-S clusters repair, which is important for cell survival.

**Figure 6:**
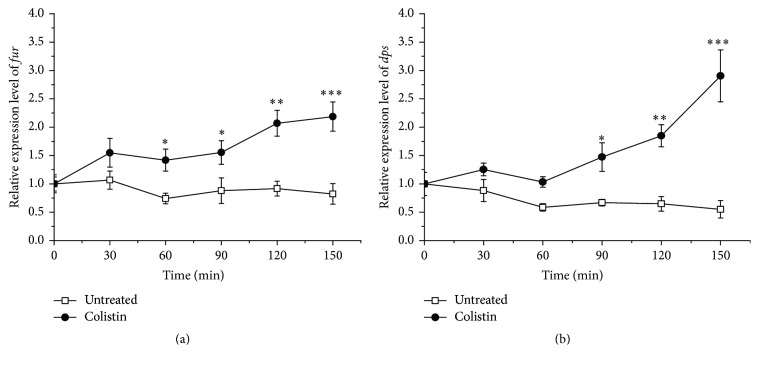
Effect of colistin on relative expression level of* fur* (a) and* dps* (b) encoding for Fur-like protein and DNA-binding protein from starved cells (Dps), respectively, in* P. polymyxa*.

**Figure 7:**
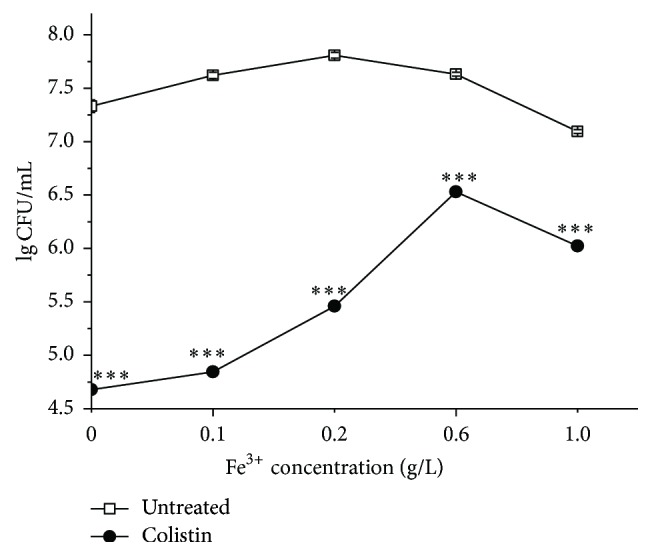
Enhancement of survival of colistin-treated* P. polymyxa* by adding FeCl_3_.

Fur-like protein (Fur), encoded by* fur*, has been reported to regulate Fe^3+^ assimilation with defense against oxidative defense [36]. Besides, DNA-binding protein from starved cells (Dps), encoded by* dps*, has been found to prevent DNA cleavage from oxidative stress by sequestering Fe^2+^ [37]. We next sought to investigate the differentiated expression of* fur* and* dps* in* P. polymyxa* cells when exposed to colistin. The data in Figure 6 show that the relative expressions of both* fur* and* dps* from colistin-treated cells appear to extraordinarily increase relative to the colistin-untreated ones. In addition, the extent of elevated expression of both genes is positively associated with the treatment time. Most probably, to survive, the colistin-exposed cells increase the expression of* fur* and* dps* for iron uptake and sequestering, thus suggesting that Fe^3+^ assimilation plays a critical role for cells to defense with colistin-induced oxidative stress. Therefore, we next sought to directly block the harmful effects of colistin-induced oxidative stress by adding Fe^3+^ to drug-treated cultures. It was found that cultures treated with colistin and Fe^3+^ show a significant delay in cell death and particularly a near 1.8-log increase in survival at 0.6 g/L Fe^3+^ relative to colistin treatment alone (Figure 7), demonstrating that Fe^3+^ is able to mitigate bacterial cell death following colistin treatment. On the other hand, there is a slight drop in LgCFU/mL at 1.0 g/L Fe^3+^, which is most probably due to slight toxic of Fe^3+^ at high concentration to cells as shown in colistin-absent treatment.

## 4. Discussion

With the alarming spread of antibiotic-resistant strains of bacteria, a better understanding of the specific sequence of events leading to cell death by colistin is needed for future drug advancement. As a cyclic lipodecapeptide, colistin carries five free amino groups with five positive charges. It was reported to specifically kill Gram-negative bacteria through membrane lysis by targeting negatively charged LPS and enhancing the permeability of bacterial membrane [4]. In contrast, Gram-positive bacteria are typically believed to be resistant to colistin due to its lack of abundant negatively charged LPS [23, 38]. It is somewhat surprising that current in vitro work has shown that colistin can also kill Gram-positive bacteria [10, 21, 22], but the bactericidal mechanism is not very clear yet. Recently, it was found that the three major classes of bactericidal antibiotics including *β*-lactam, aminoglycoside, and quinolone, regardless of predominantly well-known drug-target interaction, induce the harmful ^•^OH formation in bacteria and cause redox-related physiological alteration and toxic metabolic perturbation, ultimately resulting in cell death. Therefore, ROS formation induced by bactericidal antibiotics was proposed as a common mechanism of bacterial death [12–15]. More recently, it was reported that colistin, regardless of common membrane lysis, can also induce ^•^OH production for rapid killing of Gram-negative bacterial cells [11]. In this study, we, for the first time, have shown that colistin can kill* P. polymyxa*, a Gram-positive bacterium, also by inducing ROS. ROS accumulation results in oxidative damage including DNA break, lipid peroxidation, and downregulation of gene expression for Fe-S cluster protein (Figure 2), which is lethal to cell. The bactericidal activity of colistin against* P. polymyxa* is both dose- and course-dependent (Figure 1). ROS scavengers including thiourea, cysteine, and glutathione all yield significant increase in* P. polymyxa* survival following addition of colistin (Figure 3), confirming that colistin-induced ROS is involved in colistin-mediated* P. polymyxa* cell death. Among these three scavengers, thiourea is the most efficient at mitigating cell death within 2 h following colistin treatment, which is reflected by the most capacity of thiourea to increase LgCFU/mL close to the colistin-absent one. Since thiourea is generally believed to specifically target ^•^OH [12], these results indicate that colistin-induced ^•^OH formation via the Fenton reaction appears to be the most significant contributor to Gram-positive bacterial cell death among the formed ROS.

Our understanding of the bacterial responses that occur as a consequence of colistin treatment remains incomplete. In this study, we found that colistin treatment could increase relative expression of* fur* encoding for Fe^3+^ import regulator Fur (Figure 6(a)), which is in line with the stimulation of Fe^3+^ assimilation (Figure 5). Fur itself is a mononuclear iron protein and tends to lose activity under oxidative stress, which could potentially lead to derepression of iron acquisition system and stimulation of iron import [37]. It is important to note that there is no detectable intracellular Fe^3+^. Therefore, the assimilated Fe^3+^ could be used to form Fe-S cluster for repairing the oxidative damage, thus providing significantly protective effect against colistin killing (Figure 7). On the other hand, the assimilated Fe^3+^ could convert to Fe^2+^ for Fenton-mediated ^•^OH formation by colistin. However, both extracellular and intracellular Fe^2+^ are undetectable when exposed to abundant Fe^3+^ up to 0.8 g/L following addition of colistin (Figure 5), suggesting that not the abundant external iron import but the intracellular oxidative damage of Fe-S clusters as a key source of ferrous iron drives the ^•^OH formation, which is in agreement with the previous study [12]. In addition, colistin treatment also stimulates the relative expression of* dps* encoding for Fe^2+^ sequester (Figure 6(b)), which is controlled by OxyR regulon [39] and probably a key source of Fe^2+^ shielding from Fenton chemistry for ^•^OH reduction. Mn-SOD (encoded by* sodA*) is known to be activated by SoxS transcription factor [16, 39]. As the first responder in protecting O_2_^−^-induced damage, the* sodA* expression was significantly upregulated following colistin exposure (Figure 4(a)). In addition, the relative expression of Fur-regulated* sodB* encoding for Fe-SOD fluctuated increased under colistin (Figure 4(b)), which is in accord with the fluctuation decrease of transcript levels of DHAD (Figure 2(d)), thus illustrating that Fe-SOD is important for protecting cytoplasmic enzyme-DHAD from metabolic oxidative damage when exposed to colistin. To survive in colistin exposure,* P. polymyxa* cells have to adjust physiological processes to avoid threatening from oxidative stress [39]. Under control of SoxS regulator, Mn-SOD was activated to remove O_2_^−^. OxyR system stimulated Fur synthesis and thereby controlled the Fe^3+^ import to repair the damaged Fe-S clusters. Meanwhile, OxyR-mediated Dps expression alleviated the toxicity of Fe^2+^ from Fe^3+^. Subsequently, the activated Fur stimulated the Fe-SOD upregulation to protect DHAD.

**Figure 8:**
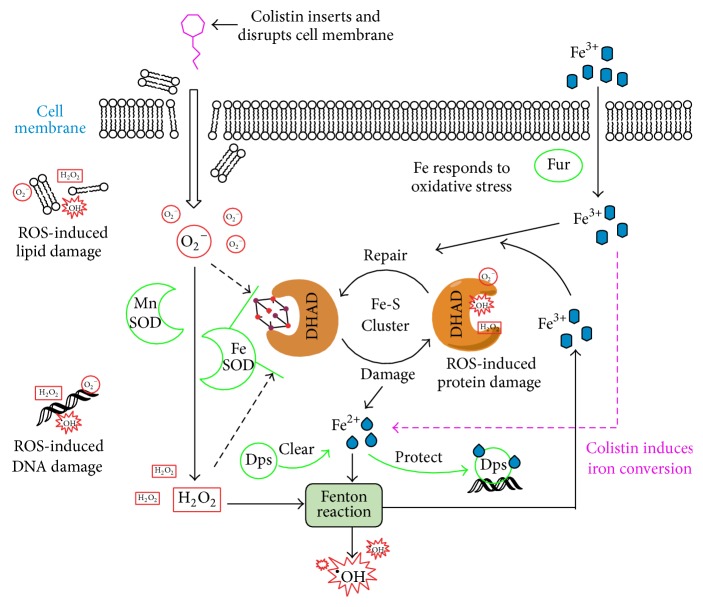
Lethal mechanism in Gram-positive bacteria caused by colistin-induced ROS [23].

In conclusion, our results showed that colistin can possibly induce rapid cell death through induction of ROS formation (Figure 8), a result of the oxidative damage of DNA, lipid, and Fe-S protein (DHAD) due to colistin in* P. polymyxa* [12, 23, 38]. It has been hypothesized that O_2_^−^ will be induced when colistin enters into and cross cell membrane [12]. Then, O_2_^−^ will be converted to H_2_O_2_ by Mn-SOD, and Fe-SOD can protect DHAD from O_2_^−^ and H_2_O_2_. As a response, Fe^3+^ will be assimilated into cells with the help of regulator Fur for repairing the inactivated Fe-S cluster. The released Fe^2+^ from Fe-S cluster will participate in Fenton reaction to produce Fe^3+^ and ^•^OH (one of ROS). In this Fe-S cluster redox cycling, iron conversion is induced by colistin. Besides, Dps can be used to isolate Fe^2+^ for protection of DNA. Therefore, ROS accumulation and oxidative damage can be induced by colistin in* P. polymyxa*. Meanwhile, cells will accelerate Fe^3+^ assimilation and use ROS scavenging system to respond to colistin-induced oxidative stress for survival.

As an essential metal in biology, iron, especially in Fe-S cluster, is thought to be one of the earliest iron cofactors, playing an important role in responding oxidative conditions [40]. In this study, we mainly expounded the role of colistin in inducing oxidative stress and iron in repairing oxidative damage in* P. polymyxa*.* P. polymyxa* is the producer of colistin. Therefore, understanding of the bactericidal mechanism of colistin against its producer by inducing ROS formation would not only enrich our knowledge of colistin against Gram-positive bacteria but also provide an important guideline based on removal of ROS damage and addition of Fe^3+^ for optimization of fermentation condition and improvement of colistin output in the future.

## Figures and Tables

**Table 1:** Primers used for gene amplification in the PCR.

Gene^a^	Forward sequence^b^ (5′ - 3′)	Reverse sequence (5′ - 3′)
*sodA*	GGCATTTCAATTACCAGAAC	GCAGCCGCGTAACGTTTGTT
*sodB*	ATGCTGAGTACTTATGGGTCTTTCC	TTAGAAGGGTTGCCATCTCAGC
*ilvD*	GCGGTCCTATGAAAGCTGGT	CGTTACTTGCGTTCGTCACC
*Fur*	CCGTGGAAGTCCAAACGATG	GCAATCCCAGGGCTACAAAC
*Dps*	AACCGTCAAGTCGCTAACCT	CCCAAATAAGAGCGCAGCATC
16S rRNA	AGAGTTTGATCCTGGCTCAG	ACGGCTACCTTGTTACGACTT

^a^The  *sodA*, *sodB*, *ilvD*, *Fur*, and *Dps* encode Mn-superoxide dismutase (Mn-SOD), Fe-superoxide dismutase (Fe-SOD), dihydroxy-acid dehydratase (DHAD), Fur-like protein, and DNA-binding proteins (Dps), respectively.

^b^The primers for different genes were designed based on the reference gene sequences of *P. polymyxa* E681 (GenBank accession number CP000154) and *P. polymyxa* SC2 (GenBank accession number CP002213), and the primers of 27F and 1492R were used for 16S rRNA gene.

**Table 2:** Primers used for quantitative real-time PCR.

Gene	Forward sequence (5′ - 3′)	Reverse sequence (5′ - 3′)
*sodA*	CCAATCTGGACAGCGTTCCT	CGCCGTTAGGAGCGATAACT
*sodB*	GGGAGTCTATTCCGCTGCTG	TGACGACATGCCACCAATCT
*ilvD*	CAATGAAGTCGCGAACCGTG	CATTCAGCACTGCGCTTACG
*Fur*	CTCCTCTAACGGTCCAAGCC	TATGATTTGCGCGGCGATAC
*Dps*	AAGGTTCTCCTTCCGCAACA	CCTCAATCAGGGTTTGCACC
16S rRNA	GAGAAGAAAGCCCCGGCTAA	ACCAGACTTAAAGAGCCGCC
